# Visioning an Effective Health Encounter: Indigenous Healthcare Experiences and Recommendations for Health Professionals

**DOI:** 10.3390/ijerph20206917

**Published:** 2023-10-13

**Authors:** Melissa E. Lewis, Sky Wildcat, Amber Anderson

**Affiliations:** 1 Department of Family and Community Medicine, University of Missouri School of Medicine, Columbia, MO 65201, USA; 2 Department of Higher Education, College of Education and Health Professions, University of Arkansas, Fayetteville, AR 72701, USA; 3 Department of Health Promotion Sciences, University of Oklahoma Health Sciences Center, Oklahoma City, OK 73104, USA

**Keywords:** Indigenous health, healthcare delivery, provider bias

## Abstract

Purpose: Indigenous patients experience challenges while accessing and utilizing healthcare services that relate to worsened health experiences. Bias towards Indigenous patients is prevalent in healthcare settings and leads to poor health outcomes. The purpose of this study was to learn about the healthcare experiences, both positive and negative, of Indigenous patients and solicit subsequent recommendations to improve care delivered to this population. Methods: This study sampled Indigenous patients (*n* = 20) from an Indigenous-serving health clinic to discuss participants’ health experiences and elicit recommendations for improved care. Four focus groups were conducted, and template analysis was employed to analyze the data. Results: A total of 15 themes were developed under the category of an effective health encounter. Highlighted themes include healthcare that is free of stigma, quality care, respecting trauma experiences, expanded integrated care and the patient–provider relationship. Based on participant recommendations, a checklist was created for healthcare professionals to improve care delivery to Indigenous patients. Results indicated that bias in healthcare settings may masquerade as poor clinical care but is really founded in biased beliefs and healthcare delivery. Alternatively, when patients received good quality care, their healthcare outcomes improved. Further, effective healthcare incorporates culture, family, tribe, and community and addresses these aspects of health in both clinical and systemic settings. Conclusions: With some of the largest proportions of health disparities and bias experiences in the US, it is critical that healthcare delivered to Indigenous patients incorporate culturally safe care to regain dignity and improve health outcomes for this population.

## 1. Introduction

Indigenous people across the United States have some of the highest rates of physical and mental health distress [1] and illness [2]. Policies of forced assimilation and cultural genocide since European contact have disrupted Indigenous lifeways and positive health, contributing to high rates of disease today [3]. Though health disparities within Indigenous communities vary greatly by community and region [4], Indigenous people suffer from a lower life expectancy [5,6,7], high infant and child mortality [8,9], high maternal morbidity and mortality [10], high rates of cardiovascular disease [11], substance abuse [12], depression [13], and suicide [14,15]. Artifacts of colonization continue to affect Indigenous people today in the form of bias and racism [16]. Further, Indigenous people have their own effective systems of health and health beliefs that are critical for providers to respect and utilize in their practice [17,18].

Indigenous people experience high rates of discrimination in a variety of healthcare settings. Compared to non-Hispanic white patients, Indigenous people are ten times more likely to experience discrimination [19] and are 2.6 times more likely to report racially biased maltreatment [20]. Ortega and colleagues (2012) found that wait times for Indigenous children was longer than any other racial groups, and only 58.3% of American Indian children received opioid-containing prescription compared to 67.4% of white children [21]. In a study of implicit and explicit bias towards Indigenous children and their caregivers in the emergency department (ED), 84% of providers had a preference for non-Hispanic white patients [22]. Further, Indigenous children were viewed as difficult and their parents or caregivers as less compliant compared to non-Hispanic white parents [22]. In another study of implicit bias, providers held a biased belief that Indigenous patients are non-compliant [23]. However, those providers with a history of bias training or cultural competency had reduced Indigenous-specific bias.

The effect of discrimination on Indigenous patients in healthcare settings carries serious health risks. Healthcare professionals note that Indigenous patients experience unwelcoming healthcare environments, stigma and stereotyping and, finally, clinical practice informed by racist beliefs [24]. Indigenous patients report being “treated differently” in health settings due to their race. They describe being denied pain medication because of false “drug-seeking” narratives [25]. Patients were also sometimes discharged without treatment or denied safety and discharged in the middle of the night. One participant described discrimination within hospital policies that kept mothers from smudging* their babies.

FOOTNOTE: Smudging is a practice used by many, but not all, Indigenous communities to cleanse or protect by burning select plants and moving the smoke over a person or object. Plants and protocol vary by community.

Given these discriminatory experiences in healthcare settings, Indigenous patients are more likely to avoid healthcare and miss crucial prevention and treatment appointments to avoid experiencing racism and bias. Nearly one in six American Indians avoided healthcare for either their family members or themselves because of anticipated unfair treatment or discrimination [26]. Further, Indigenous patients who have experienced discrimination by a healthcare provider are more likely to express experiencing an unmet health need within the previous year [27]. Finally, patients who report increased experiences of discrimination in healthcare settings were less likely to be current on critical cancer prevention care, putting them at risk of developing preventable and deadly forms of cancer [28].

Director of the Center for Disease Control, Dr. Rochelle Wolensky, recently said that “racism is a serious public health threat that directly affects the well-being of millions of Americans. As a result, it affects the health of our entire nation” [29]. Specifically, bias and racism towards Indigenous patients are prevalent within healthcare settings and relate to worse health outcomes [16]. Discrimination within healthcare settings can have negative effects on Indigenous people, such as being denied proper treatment or avoidance of critical preventive visits to avoid racist experiences [25]. With the high rates of discrimination and its concerning health effects, more information is needed to inform healthcare professionals about the needs of Native patients within healthcare settings from their own viewpoint.

This study aims to elicit Indigenous patient experiences of healthcare, as well as recommendations to improve care. This study provides a valuable contribution to the literature by elevating pertinent Indigenous perspectives and providing patient-derived recommendations on how to conduct an effective and culturally safe health encounter for Indigenous patients. Further, these experiences informed the development of an applicable toolkit to improve the effectiveness of healthcare encounters across a multitude of Indigenous populations that is being piloted and tested at several sites.

## 2. Methods

### 2.1. Study Purpose and Research Questions

Widespread healthcare discrimination and significant disparities in physical and mental health outcomes for Indigenous people underscore the pressing need for improvements in the delivery of healthcare services to this population. To inform said improvements, researchers conducted a qualitative, descriptive study utilizing focus groups rooted in Indigenous Research Methodology (IRM) to answer the following research question from the perspective of Indigenous patients: (1) What is the experience of Indigenous patients in healthcare settings? And (2) What changes are recommended to improve healthcare encounters?

### 2.2. Method

Four focus groups were conducted at an urban, Native American-serving health clinic in the upper Midwest US that provides medical, behavioral (individual and group therapy for mental health needs and substance abuse), and dental services to patients. Participants were recruited at the clinic post-appointment to complete a survey and participate in a focus group later. Flyers were given to all participants seen at the clinic, and researchers were on site to direct participants to take the survey in a private room. Researchers employed purposive sampling strategies to recruit individuals who met the following study inclusion criteria: adult (age 18 or older), Indigenous (self-ascribed), and were seen at the clinic on the day of recruitment to ensure the recent use of healthcare services. Twenty people completed informed consent and participated in the focus groups. Four meetings were held within a two-week period with the following participants in each group: 3, 5, 3, and 9. Participants were asked about their healthcare experiences broadly and concerning the training that they would like their healthcare providers to have. It is important to note that participants vacillated between sharing their healthcare experiences at the clinic that they were recruited from and other sites. We did not demarcate which health site participants were referencing. All focus groups were audio-recorded, and audio recordings were subsequently transcribed by a third party prior to analysis. Focus group questions can be found in Appendix A. This study was approved by the University of Missouri Institutional Review Board.

### 2.3. Analysis

The study authors are three Indigenous (enrolled citizens of tribal nations) women from the lower Midwest US who conducted a template analysis [30] of the transcribed focus group content. Authors first read through the transcripts several times each to familiarize themselves with the data. Then, they all met to develop a list of candidate themes to be applied during coding. Once candidate themes were identified, all three authors began jointly coding the data, discussing any disagreements, and arriving at consensus on all coding decisions. After the authors completed coding the full data set, they defined an initial template that summarized the themes they had identified that addressed the study aim. This template was applied and modified as needed for the remainder of the data analysis, resulting in a final template (see Table 1) that summarized and defined identified the themes.

The entire template included 7 themes:

1. Ineffective health encounters;
2. Effective health encounters;
3. Improvements needed for healthcare encounters;
4. Systemic and structural barriers;
5. Effective healthcare systems;
6. Improvements needed for healthcare systems;
7. Indigenous knowledge and beliefs.

These themes are presented in a previous manuscript (author citation). Due to the richness of one theme in particular, “Effective Health Encounter”, as well as the importance of the topic, the authors decided to present this theme alone for the purposes of this manuscript. The data collected and analyzed concerning this theme are important because they share critical Indigenous perspectives of healthcare and proposed solutions.

### 2.4. Indigenous Research Methodology (IRM)

IRM is based on the fundamental belief that knowledge is relational and shared with all of creation; this means that the relationship extends from not only interpersonal relationships or with research subjects but also with the cosmos, animals, plants, and the earth [31]. Relationality is at the core of how scholarship and research are engaged. The authors used IRM as a framework for project creation, data collection, and data analysis. Specifically, the researchers engaged with clinic administration, staff, and elders advisory board concerning data collection and dissemination. We completed a retreat at an Indigenous center catered by Indigenous chefs and spent an entire day discussing the research project with key stakeholders. The researchers disseminated data and analysis multiple times to the clinic staff and elders for feedback and direction toward the next steps. Finally, all authors are Indigenous, utilize IRM in their work, and worked to prioritize IRM at each analysis meeting. Further, one theme was identified as Indigenous ways of being that coded notable quotes demonstrating Indigenous-specific ways of relating, responding, or communicating that will be discussed further in a subsequent manuscript.

## 3. Results

### 3.1. Participant Demographics

Demographics were collected from 20 participants (see Table 2). All were Indigenous, with the majority identifying their tribal affiliation as falling into the broad categories of Anishinaabe (*n* = 15) and others identified as Lakota (*n* = 5). There were more women than men or gender-neutral participants (16 women, three men, and one gender-neutral), and the average participant age was 46.25 years. Incomes ranged from USD 0 to USD 37,000, with USD 11,709 as the average yearly household income. Participants indicated if they suffered from physical health illness (70%) (e.g., diabetes, seizure disorder, fibromyalgia), behavioral health illness (70%) (e.g., depression, anxiety, post-traumatic stress disorder), and substance use (50%) (e.g., alcohol, methamphetamines, opiates). Finally, three participants indicated that an Indigenous language was their first language.

### 3.2. Results

The researchers identified a total of 15 sub-themes that described Indigenous patients’ descriptions of effective healthcare encounters that summarized their recommendations for improvements in healthcare encounters. All themes are defined, along with illustrative quotations from study participants, in Table 2. For brevity, five of the most salient sub-themes are described in detail below. These five sub-themes represent the most frequently coded of all sub-themes.

A checklist was created to succinctly summarize participant needs and recommendations for experiencing an effective health encounter (see Table 3). This checklist is based on the concerns and preferences noted by study participants. It is meant to be a starting point for providers to explore the needs of their patient population through their own personal exploration, education, and growth, attendance of community events, and Indigenous mentorship, for example. This list does not fully encompass all needs of Indigenous patients nor provide tribal or geographic needs for all Indigenous patients. We hope it will be tailored and localized, piloted, and refined at multiple sites and with a variety of communities.

Participants shared their healthcare experiences, both negative and positive, resulting in a picture of the care they would like to receive in the Effective Healthcare Encounter Theme with 15 sub-themes identified and, for brevity, 7 are described in detail below. This vision of healthcare was Indigenous-specific and bias-free. Further, participants shared that when they received good care (as described in the results below), it resulted in improved wait times to see a provider, patient trust of the provider and clinic, patient–provider relationship, medication adherence, health outcomes (e.g., reduction in A1c levels), and eating habits.

### 3.3. Free of Stigma

Indigenous people experience high rates of discrimination throughout their lifespan, and discrimination in healthcare settings is no exception. Specifically, Indigenous participants noted that they experienced stigma around their race, as well as illnesses that are stereotypically associated with being Indigenous. One participant contextualized healthcare discrimination through an early life experience: “And when we were all growing up, all of us here at the table, in the textbooks we read bad things about Natives. So that’s (how) they (non-Native people) are raised in school. When they came to America, Native people were bad and they had to fight them all the time. But we were…not like that, and today that’s what is still in the books. [Several affirmative yes or umm hmms.] That’s why we’re lucky to have teachers coming forward, Native teachers, to really teach us our ways and all these good activities and events. Teachings are going around the neighborhood that you can join.” This participant describes the origins of the teachings of healthcare providers and implies how this early childhood learning experience not only negatively affects Native people by being treated as if these false stereotypes are true, but it also sets the stage for those who are going to become healthcare providers to have biases against Native people in healthcare settings. This participant also reflects happiness over the revitalization of Indigenous lifeways in the community.

Other participants reported feeling stigmatized in the context of addiction. One of the most prevalent false stereotypes about Indigenous people is that they abuse substances, and there is evidence to suggest that these biases relate to worse care [21,22]. Patients described that bias-free care looks like good quality clinical care. For example, “When I was going to the doctor before I came here, I wanted to get help and it was very hard. As soon as they find out that you’re needing help, you’re no longer a client, you’re an addict. Every place I went before they would say, ‘no it’s a waiting list’ and they don’t give medicine to help people to help them to withdraw. Here, the same day I got medicine and they brought me in and made sure I was comfortable, I was really in bad shape. They [providers] really took personal time to help. We’re not drug addicts, alcoholics, we’re regular people we just can’t help it.” This participant painted a picture of how bias affects clinic policy, clinical decisions, and clinical outcomes. At the Native serving clinic, participants felt they were treated “nice” and given proper care.

### 3.4. Quality Care

Participants reported receiving better quality of care and reduced bias at a Native-serving clinic compared to other clinics. Quality care included meeting the minimal standard of care in three areas: thorough assessment leading to correct clinical decision and treatment plan, patient understanding of illness and treatment plan, and follow-up care to assist with medication adherence and carrying out the treatment plan at home.

A thorough evaluation is an example of the care that a patient receives when it is free of bias. Participants reported feeling surprised and happy when they received basic quality care, including proper assessment, proper explanation of medications and treatment plan, given they had not received this type of care often or at all. Participants associated thoroughness in assessment with provider competence, which bolstered the patient’s belief that the provider was a skilled and caring provider.

Inadequate assessment can lead to misdiagnoses. Participants pointed out that they shared information with providers that went ignored and ultimately contributed to misdiagnosis:

“What I really disliked was that the doctor did not trust my mother’s intuition when it came to my child and she pretty much dismissed what my thoughts and feelings were and… he went into a full blown seizure after that. That frustrates me because I’m the mother. I live with this kid 24/7 and just because you’re a professional at a doctor’s level you won’t take in consideration of what others see and what they know?” Another participant shared their experience; “It’s like when I kept having a bad headache here and the day I came, they didn’t know I (was having) a stroke.”

Alternatively, good clinical decisions were described as ensuring that patients understand their treatment plan and medications, providing opportunities for feedback on treatment plans and providers, ensuring patients are satisfied with the way information was provided to them, and timely follow-up contacts and routine updates of their care plan. “All that kind of basic stuff. Everything’s explained to me thoroughly. And she makes sure that I understand the medications they’re putting me on, and how to take them or what I’m using this medication for.” This participant shared that they were satisfied with care in which the provider explained medications thoroughly and noted that this was the “basic stuff”, meaning that a minimal amount of care is enough to feel satisfied.

Finally, participants reported feeling cared about because their providers contacted them after visits to ensure they were all right. A participant expressed that a positive patient–provider relationship included keeping up contact and following up on patient needs, resulting in effective care even when the physician may not have been competent in Indigenous cultures: “I had a positive experience with my doctor. He’s not culture based, he’s white, I guess. I have had him since I was about 16 and he knows about my addiction and he has follow-up questions, like during his office hours he calls and checks and says, ‘hey I got your test results’, anything to inform you about your care plan or whatever. Like I think, I hear a lot about how there’s not good communication so that would be good for your team to have. Like to reach out to your clients in your care would be nice too.”

### 3.5. Respecting Trauma Experience

Participants reported that the intersection of chronic health needs and a history of trauma makes standard medical assessment and treatment challenging, resulting in patients avoiding healthcare centers altogether. Positive and supportive reactions by providers to their patients revealing or struggling with trauma experiences coupled with training in historical trauma were valued by patients. Examples of providers properly assessing and treating patients using a historical trauma lens include the following; “this is at the dental (clinic) where I deal with severe childhood trauma when it comes to sexual abuse and I can’t have anything close to my face”. This participant went on to share that the providers respected the patient’s needs and that the future providers were aware of her sexual trauma via reading clinical notes, so they did not have to repeat their trauma story for each provider, which was noted positively by the participant.

Participants explained that healthcare providers who work with Indigenous patients must learn the relationship between trauma, mental health, and physical health and be able to incorporate a proper assessment and treatment plan to address these needs. Proper assessment and treatment of trauma may result in improved health outcomes. One patient describes an example of talking with their provider about intergenerational trauma: “I know my doctor-we talk a lot and one of the things that I’m really beginning to understand is like the intergenerational trauma and how that all goes down, comes down from generation to generation because we were talking about alcoholism and I was just telling him that that was all I saw growing up, the drinking and, but it was just the normal thing to do, and how that just ties in with all that trauma and stuff”.

### 3.6. Expanded Integrated Care

Participants expressed a preference for integrated care clinics over others that are siloed for the convenience of the “one-stop shop” (medical, dental, behavioral health, and substance abuse treatment). However, successful Native-serving integrated care clinics expanded on these traditional categories of integrated care (integration of behavioral health and/or dental into medical clinics) into other socio-cultural realms, resulting in providing transportation, food at clinic, Traditional Indigenous Medicine*, trauma-informed care (e.g., dentists providing care standing up because of unique patient emotional and medical issues), healing and resilience centered engagement, and stigma-free patient recruitment techniques (e.g., substance abuse treatment information at bus stops). Further, patients reported another ecological realm of integrated care was the community itself and viewed the clinic as, “part of the community”.

FOOTNOTE: Traditional Indigenous Medicine refers to the Indigenous health practices used by Indigenous communities for centuries.

One example of how holistic interventions result in improved assessment, treatment, and treatment adherence is detailed in this participant’s story: “I’m not embarrassed to say it, so I’ll just say it, I came because I’m addicted to opiates and I seen the sign up on the bus stop and it’s hard to go to the doctor and explain I wanted to detox and then I saw the sign at the bus stop and it said to call. So, I came here and saw someone and they started a treatment plan and changed my prescription that night. I actually changed my primary to [provider here] instead of where I was before. She acts because she cares. She’s easy to talk to and you know I feel very satisfied and happy with the treatment plan. You know I was just another number at the other place. It’s great you know and they don’t treat me like a drug addict. And getting help and I see a counselor and see a therapist. I got to come here every day but they got to arrange for me a cab every day and I’ve never had anyone offer to do that for me. And from (my home city) it’s an hour and a half to get here. They arrange for a cab ride to and from. I’ve never had anybody treat me that well. And they’re always feeding me, I mean they always have food here. I’m very satisfied with the treatment. With everybody, the therapist she’s always calling, it’s been great. I would recommend this to anybody. It’s great. I know they care about me.”

Expanding integrated care for Indigenous patients includes Indigenizing all aspects of the care that is given. One participant explains that they see this occurring through such examples as updating assessment paperwork for Indigenous language or care preferences and providing Indigenous staff and interpreters: “We are the original people here at least on this continent. So, I would appreciate it if they would at least get to know our cultures and the background. They have special languages and interrupters for other people, why can’t (we) have the same thing…Like native staff, maybe have a liaison on staff, just make somebody feel comfortable and maybe sit in in case there’s no native doctor.”

### 3.7. Patient–Provider Relationship

Participants shared that they had increased satisfaction with care when they had a good relationship with their provider. This included a positive interpersonal relationship, a positive relationship with the patient’s family, a positive relationship with the patient’s Native community, and helping patients realize their potential in becoming healthcare providers. Participants also shared that aspects of a good relationship include humor, cultural humility, and genuine care for people (cultural humility is a lifelong critical analysis of one’s own lens requiring humility as opposed to knowledge or expertise [32,33]. This approach aims to address the source of bias and reduce hierarchies between others.).

Participants explained that feeling cared for in healthcare settings occurred when there was no stigma or bias, and wherein they received good quality care, they got their healthcare needs met, they felt supported and not alone in the illness, and that they were listened to and understood. One participant explained that feeling cared for meant not feeling like they were alone or like someone gave up on them: “They are the only doctors who never gave up on me. They were always there. Even when we didn’t know what was going on, they were just managing whatever was happening to me.” It is important to note that even when providers were not able to properly assess or treat the patient, this participant felt cared for by providers’ continued efforts to treat them.

One participant described why they were satisfied with the care they received, “she was really understanding and nice and was real accommodating in helping us get what she (patient) needed.” This participant noted that they were happy with the care that they received and explained that the provider demonstrated empathy and understanding and also provided relief from the distress of the illness.

Indigenous people face discrimination in society and in healthcare visits, making clinics and hospitals unsafe places, reducing care visits, treatment adherence, or trust in the provider or system. However, care and positive patient–provider relationships built over time can result in positive health encounters: “Usually we (Native people) don’t talk about our personal lives or our family. The first time I saw my physician here she seemed so comfortable, and I was able to talk to her, of course, I end up crying. It’s not easy to get used to.” A healthcare visit can be an emotional and stressful place for Indigenous people. Participants noted they often receive judgement and criticism in clinical encounters but they preferred positivity and praise from their providers: “She (provider) was glad when the lab (tech) came and gave her the results for that test and everything and she was like ‘All right!’, gave me like a high 5., so that’s good”. Positivity and support related to patient’s reports of feeling cared for and satisfied with treatment.

## 4. Discussion

In this study, patients were asked to reflect on the care they received, both good and bad, and envision the care that they would like to receive. This manuscript utilized participants’ shared needs and experiences in healthcare settings to provide direction to providers to improve the care that they deliver to Indigenous patients. A quick guide for providers was created to summarize participants’ recommendations and needs, as well as a table summarizing each theme.

The results included five highlighted themes and ten additional themes. Connected principles of all themes included the historical context of colonization, its effects on modern-day bias experiences, and the need to decolonize communities and healthcare systems. Participants felt that given that they are the original people of the continent and that they have suffered through colonization and genocide, they are deserving of culturally safe and effective care. At the same time, stigma is well documented in the general population for its harmful effects on an individual’s health and help-seeking [34]. Indigenous patients’ experience of bias and stigma intersected within the realms of race, disease type, and disability, seemingly compounding the stress and burden of bias [35]. Bias within healthcare systems results in reduced help-seeking, worsened health status, and worsened mental health, yet health systems aimed at reducing bias towards Indigenous patients result in improved health outcomes for this population [36].

Participants noted that biased care looks like poor clinical care. When a provider has a false and biased belief about Indigenous people and acts on these beliefs, it can result in poor quality of care. The Agency for Healthcare Quality (AHRQ) noted six domains of healthcare quality: safe, effective, patient-centered, timely and efficient, and equitable [37]. While equity is listed in a separate domain in the AHRQ aims, in our study, it appears that lack of equity could be elevated given that it is critical in attaining the remaining five aims. For instance, healthcare quality was negatively affected when providers dismissed the patient’s needs, were not timely, and conducted incomplete or rushed assessments, resulting in incorrect diagnosis and treatment. Finally, participants saw their cultural beliefs and practices as healing practices that they would like to see incorporated into their care. They also requested involvement in decision making around policies and systems that affected their care.

Culturally appropriate care includes care from healthcare providers who are knowledgeable about historically traumatic events that affect the health and well-being of their patient population and have positive regard for Indigenous health beliefs. Providers who assess and treat trauma artifacts, as well as trauma along the lifespan, are more likely to provide care that results in improved health outcomes. It is important to link trauma assessment and treatment with an active promotion of Indigenous culture within a clinic, such as adding Indigenous language in assessment and in signage, including Indigenous patients on boards, and updating policies to allow the use of Traditional Indigenous Medicine (TIM) and foods in hospitals, for example. Tailored and improved assessment can lead to improved provider satisfaction as well. With improved provider satisfaction, provider burnout is reduced, which discontinues the vicious cycle of biased and reduced quality of care [38]. Coupled with learning about local Indigenous communities, healthcare providers can shift their communication style, beliefs and understanding, as well as behaviors, to provide improved care to Indigenous patients [39]. Further, many models of Indigenous health and well-being already exist and can be utilized by healthcare providers in corresponding regions for assessment, treatment planning, and training. They include the Māori models of Te whare tapa whā [40] and the MIHI model [41], First Nations Mental Wellness Continuum Framework [42], Alaskan Native qasgiq model [43], Diné (Navajo) Hózhó Wellness Philosophy [44], and the Native Hawaiian conceptual model of NĀ Piko ‘Ekolu [45], for example.

It is important to note that Indigenous people experience disproportionate barriers to full and healthy lives due to systemic discrimination in education [46], housing [47], employment [48], and food systems [49], for example. These experiences relate to increased risk for chronic and acute illnesses. Participants in this study noted that healthcare that assessed and treated the whole person, including family and community, resulted in improved health outcomes. Other clinics have taken similar approaches, such as social prescribing [50] or fully integrated care [51]. Participants also discussed the need to increase the number of Indigenous doctors [52] and other healthcare professionals such as patient navigators [53] or elders in residence [54]. Specificity to Indigenous communities is needed with these techniques, and additional resources to tailor towards Indigenous communities include educational organizations such as the Leaders for Indigenous Medical Education [55] and the Indigenous Health Committee (IHC) at the Royal College for example [56]. Each has created curriculum frameworks and content for learners to improve cultural safety for Indigenous patients.

## 5. Limitations

This study had several limitations. To start, focus group size varied by group, which could lead to different group dynamics and, possibly, responses within each group. Also, focus groups took place within a clinic versus a neutral, non-clinical location, which could have limited participants’ critical opinions of their healthcare experiences due to confirmation and/or social desirability bias. Researchers worked to address this possibility of bias by sharing in the informed consent process that all research staff are mandated to keep participant information private and that there are no repercussions to their medical care or otherwise for sharing their opinions.

## 6. Conclusions

In this study, participants shared their experiences of healthcare, both biased and equitable, in healthcare settings and proposed solutions from their lived experience to reduce bias and improve the healthcare disparity gap. While race is a social construction, for Indigenous people, a group commonly identified by race instead of correctly as a political status (e.g., citizens of sovereign nations), racism plays a critical role in health status and outcomes. In particular, the American Association of Biological Anthropologists (2019) states, “While race does not accurately represent the patterns of human biological diversity, an abundance of scientific research demonstrates that racism, prejudice against someone because of their race and a belief in the inherent superiority and inferiority of different racial groups, affects our biology, health, and well-being. This means that race, while not a scientifically accurate biological concept, can have important biological consequences because of the effects of racism” [57].

This project identified specific needs and recommendations of Indigenous patients from a health clinic in the upper Midwest to receive non-biased, quality healthcare services. While these recommendations encompass a checklist for providers to improve the quality of care that they deliver to Indigenous patients, the checklist must be coupled with an inquiry into local knowledge and community needs. Successful intervention of these recommendations requires a critical awareness of self, history, land, power, and the privilege of healthcare providers and systems.

## Figures and Tables

**Table 1:** Final template: Theme domains, definitions, and exemplar quotes.

Domain	Definition	Exemplar Quotes
1. Free of stigma	Participants noted the difference between care that is free of stigma and care in which they are stigmatized for their race, their illness, or the intersection of them. Care that is free of stigma is quality care that effectively assesses and treats the patient to improve the illness without bias affecting the care that is given. Stigma for illness and race reduce the ability of care systems to provide quality care.	*When I was going to the doctor before I came here, I wanted to get help and it was very hard. As soon as they find out that you’re needing help, you’re no longer a client, you’re an addict. Every place I went before they would say, ‘no it’s a waiting list’ and they don’t give medicine to help people to help them to withdraw. Here the same day I got medicine and they brought me in and made sure I was comfortable, I was really in bad shape. They [providers] really took personal time to help. We’re not drug addicts, alcoholics, we’re regular people we just can’t help it.*
2. Quality care	Participants shared that an effective health encounter included a proper and thorough assessment, correct diagnosis, effective treatment with an explanation of the treatment plan, and proper follow-up care. For many participants, they have rarely received this kind of care.	*When I come to my appointments they do the whole, the whole rundown, you know, the blood pressure, weight and all that stuff. When they’re working with my lab stuff everything’s gone over with me. Medications are explained what they [U] to prescribe them to me and making sure that I understand.*
3. Respecting trauma experience	Indigenous patients experience disparate rates of trauma, both historical and through the lifespan. These experiences relate to negative health outcomes and, therefore, should be properly assessed and treated by the healthcare provider.	*And the last few times I’ve had two different people that worked on my dental and they were very open and understanding. But even the second time she literally put it (i.e., patient trauma story and needs) all in the notes so that I don’t have to continue to explain myself. So that is a major plus, and that’s when I’m also bringing that it’s important that they’re trained in historical trauma. You don’t know what keeps us from coming to the dentist or what’s gonna happen or what will be triggered.*
4. Expanded integrated care	Participants expressed a preference for expanded integrated care to include care in the areas of medical, dental, behavioral health, substance abuse treatment, social needs, family needs, community needs, and cultural needs.	*I finally started taking care of my health, you know, trying to look at the health, dental, primary care.*
5. Patient–provider relationship	Participants shared that a positive relationship with their provider is important to them and included feeling listened to and cared for. A positive relationship also hinged on the provider’s relationship with their family and the community.	*Having a provider who’s sensitive with some compassion would be really great.*
6. Traditional Indigenous Medicine (TIM)	a. Participants expressed wanting providers to be more aware of traditional healing methods, such as traditional medicine, ceremony, providing food, holistic theory and care, female-based care, and smudging. b. Participants appreciated having TIM services. An elder in residence is a staff member who is familiar with Traditional Indigenous Medicine and provides services to patients, supports healthcare providers, and can be a member of the treatment team.	*a. I wish there was an option for that (traditional Indigenous medicine) instead of regular medicine. There’s just so many people hooked on stuff like Opiates now because of that. I wish they’d offer more traditional medicine before they start pushing the pills on you.* *b. Like cedar tea. I’ve never drank that before until I came to (this state). (Elder-in-residence) even told us that when we’re really having a hard time to take a bath in it. I did that once and it just felt so good, very healing.*
7. Patient agency	a. Participant control over their own body and being an active decision maker in their healthcare plan improved patient satisfaction, adherence to treatment plan, and culturally relevant care. b. A patient’s ability to have control over their own healthcare treatment was described in terms of the cultural barriers that exist in activating agency and resources to help attain it.	*a. So I’m gonna continue to come to see my primary care (doctor) here. My diabetes levels-my sugar levels would have been still high, so I take my medications now like she prescribes and it’s starting to work for me now. I felt good about it, you know, and that’s what worked for me anyways.* *b. I really like that they have [name] here, like the Elder in Residence, because you can always talk to her and she can be like the advocate. I think they should have that at every clinic cause just even her presence is really powerful. Because there are a lot of cultural barriers.*
8. Gender preference for provider	a. Indigenous women reported a preference for female providers, especially around health issues including pregnancy, childbirth, menopause, moontime (menstruation), sex, sexual abuse. Participants reported that these topics have specific cultural values that are tied to gender-specific knowledge and practice. b. Cultural practices involve rules and protocols around gender and should be respected in healthcare settings, including what gender should discuss and treat certain illnesses. For example, part of culturally relevant care is knowing that an Indigenous woman might request a female provider and respecting that, which can include having a female Native patient navigator if there are only male doctors.	*a. A lot of us, we have traditions revolving when we’re menstruating we call on our moon. So, I wouldn’t feel comfortable talking to a male, in particular a non-native male that’s not going to understand that. So, if we could have an option of a female for those kinds of visits. Like sometimes there’s issues that are sexual that we have to deal with where again I wouldn’t be comfortable with a man again especially where there’s a particular background so to speak.* *b. If there was an option to have a female native, pretty much any native woman, I definitely be more comfortable with something like that. You know going through a pap smear or things like that involve women issues, menopause, menstruation, that kind of stuff. I mean if they offered native medicine instead of trying to push a pill because they don’t help anything just mask it. So, I’d much rather use a native traditional man rather than have one with modern medicine.*
9. Native American-serving clinic	Given the lack of good care and culturally relevant care, Native people prefer to seek care at a clinic that has a mission to work with Native people, hires Native people, is in a Native community, and has providers trained to work with this population.	*Natives want to go to somewhere that’s culturally involved...people that understand that our people, our ways. It’s a good feeling going to a Native clinic.*
10. Appropriate billing practices	Participants shared experiences in which they have been refused service due to their socioeconomic status or their assumed socioeconomic status by their race.	*I took my son over there and I gave them my tribal card and they looked at me and said they would not see my son unless I had the cash or a credit card to hand to them. [Specific clinic] will bill you later and they will see your children and they will see you and they will bill you later.*
11. Continuity of care	Relationality is a key component of Indigeneity; therefore, the state of the relationship between patient–provider is a critical component of care with Indigenous populations. Participants expressed the importance of having a consistent provider that is familiar with their needs. Participants reported it was helpful to not have to repeat illness or trauma stories, long-standing relationships and knowing their family members, and this led to (increased satisfaction and adherence to treatment plans)	*She acts because she cares. She’s easy to talk to and you know I feel very satisfied and happy with the treatment plan. With everybody, the therapist she’s always calling, it’s been great.*
12. Intergenerational care	Family centered care was an important part of preferred care delivery: Participants reported going to visits with family members, sharing providers with family members, talking about family members who were also patients of their provider during appointments, and answered questions about care needs with family members in mind.	*I think it’s important, especially in developing that relationship and knowing that they actually genuinely care about my family.*
12. Native healthcare providers	Participants expressed a desire for providers who are from their community to understand their needs and challenges.	*Natives want to go to somewhere that’s culturally involved and people that understand that our people, our ways.*
13. Community familiarity	Participants appreciated providers being familiar and from the community they were serving.	*Keep treating the community that they treat because they’re in the community, so they know the people that they’re servicing*
14. Timely and convenient	From scheduling an appointment to receiving medication, participants expressed satisfaction with care when it is timely and accessible.	*You can always come, you’ll get seen. With dental, or medical even with a drop in, you will be seen. Like on the reservation if you want to see the dentist appointments will be like 2 months out but if you come here you get in right away you and be seen. Like you said, I have come all the way from [a different state] because they have all these events going on for natives and different kinds of medical and treatment approaches, the staff are always welcoming and friendly, the surroundings are comfortable, plus it’s easy access.*
15. Spending time with patient	Patients expressed that it takes time to get to know a patient and that was important to them and that this resulted in improved health outcomes.	*She just took the time to, to really listen to what I was talking about and then she came back with everything I needed, and it was good.*

Note: 1–5 are described in the text.

**Table 2:** Participant demographics (*n* = 20).

	*n*	%
Age, mean (sd)	46.25	12.65
Income, mean (sd)	$11,709.60	9746
Gender		
Female	16	80%
Male	3	15%
Gender-Neutral	1	5%
Tribal group		
Anishinaabe	20	75%
Lakota	5	25%
Reported Physical Illness		
0	6	30%
1	10	50%
2	3	15%
4	1	5%
Reported Emotional Illness		
0	6	30%
1	9	45%
2	3	15%
3	2	10%
Reported Substance Use Disorder		
0	10	50%
1	6	30%
2	3	15%
3	1	5%
First Language (*n* = 19)		
English	16	84%
Indigenous (Anishinaabe or Lakota)	3	16%
Language currently spoken (*n* = 19)		
English only	16	84%
English and Spanish	2	10%
English and Indigenous (Anishinaabe or Lakota)	1	1%

**Table 3:** Recommendations for achieving an effective health encounter with Indigenous patients.

Clinical care delivery
☐ Trained and informed in Indigeneity ○ Provider is knowledgeable of Indigenous history, Indigenous people of that region, and basic cultural beliefs and practices ☐ Acknowledge settler-colonial trauma and its effect on the health and well-being of Indigenous patients ○ Acknowledge healthcare settings as sources of trauma due to discrimination and build relationships accordingly. ☐ Provide bias and stigma-free care (e.g., race, disability, mental health, substance abuse, cardiometabolic syndromes) ○ Provide quality care. Use cultural humility techniques (e.g., Do I provide the same quality of care to all my patients? How do I know? Why or why not?) ☐ Assess and treat modern-day and historical trauma ☐ Assess Indigenous linguistic and cultural health needs and beliefs and incorporate them into a treatment plan ☐ Support Indigenous beliefs and lifeways in clinic ☐ Consider the whole patient system in a treatment plan ○ Include family (patient-defined) needs, community needs, tribal needs ☐ Consider Indigenous beliefs and resources in a treatment plan ○ Be familiar with community resources for Indigenous people (e.g., social activities, ceremonies, traditional medicine people for referral) ☐ Provide a thorough explanation of diagnosis and treatment ○ Ensure patient understanding of each ☐ Provide opportunity for patient feedback ○ Check on patient satisfaction. Did the patient receive what *they* came to the clinic for? Do they believe you listened and understood their concerns? ☐ Follow up with patients in a timely manner after the appointment. ○ Ensure understanding of diagnosis, medication, and treatment plan Patient–Provider Relationship ☐ Build a *real* relationship with your patient. Positive provider attributes include... ○ Respectful, nice, caring, understanding ☐ Allow and use humor ☐ Reduce patient–provider hierarchy by using cultural humility. ○ Acknowledge and validate the expertise of Indigenous patients over their bodies and life experiences. ○ Co-create the treatment plan ☐ Notice and praise patients for work towards and success around positive health behaviors Administrative Practices and Policies ☐ Provide timely services (e.g., appointments, medication) ☐ Provide care first, bill later ☐ Provide expanded, Indigenous-centered Integrated care ○ Care in the realms of medical, dental, behavioral health, substance abuse treatment, social needs, family needs, community needs, and cultural needs. ○ Provide Traditional Indigenous Medicine ☐ Continuity of care ○ Provide an environment that will reduce provider turnover and burnout ☐ Ability to choose the gender of the provider and/or provide a staff person of that gender ☐ Ability to choose the race of the provider and/or provide Indigenous support staff

## Data Availability

Not applicable.

## Appendix A

**Table A1:** Focus Group Questions.

Question	Response Options
Introduction: We are working to create a training for all healthcare providers at (clinic) to improve the care they deliver. We are particularly interested in discussing what is needed so that healthcare providers deliver culturally sensitive care to patients (What we are not discussing how the clinic operates: issues related to wait times, and contract health services issues for example).	
Main Question: 1. What would you like to see as part of their training to meet this goal?	Open
Follow-up Questions 2. What would you like your healthcare providers to know about you, your family, your culture to be able to treat you effectively?	Open
3. Do you think your providers know enough about Native American people and culture?	1. No. If no, what more do they need to know? 2. Yes. If yes, what things do they already know that have been helpful as they care for you?
4. Do you wish your healthcare provider would treat you/your family differently? a. Do you think you are treated differently because you are Native American? b. Different cultural/racial communication?	1. No. If no, what do you like about how your provider treats you? 2. Yes. If yes, how would you like to be treated?
5. Do you get the healthcare you want?	1. No. If no, what would you like to see more/less of? 2. Yes. If yes, what do you like about it?
Wrap-up Questions 6. Of all the topics that we discussed around your personal experiences of healthcare, what is the most important experience that came up for you?	Open
7. Is there anything that we should have talked about but didn’t in regards to your healthcare experiences?	Open
